# Self-directed passive-aggressive behaviour as an essential component of depression: findings from two cross-sectional observational studies

**DOI:** 10.1186/s12888-022-03850-1

**Published:** 2022-03-19

**Authors:** C. G. Schanz, M. Equit, S. K. Schäfer, T. Michael

**Affiliations:** Division of Clinical Psychology and Psychotherapy, Department of Psychology, Saarland University, Saarbruecken, Germany

## Abstract

**Background:**

The self-control model of depression suggests depressive symptoms to derive from distorted self-monitoring, dysfunctional self-evaluation and reduced self-reward as well as increased self-punishment. Building on this model a relationship between self-directed passive-aggressive behaviour, that is, harmful inactivity, and depression has been assumed. This association has been supported by a recent study in an inpatient sample. However, it remains unclear if patients with depressive disorders report more self-directed passive-aggressive behaviour than patients without depressive disorders and if self-directed passive aggression mediates the associations between distorted self-monitoring and dysfunctional self-evaluation with depressive symptoms.

**Methods:**

Study 1 compared self-directed passive-aggressive behaviour levels between 220 psychotherapy outpatients with (*n* = 140; 67.9% female; *M*_*age*_ = 40.0) and without (*n* = 80; 65.0% female; *M*_*age*_ = 36.2) depressive disorders. Diagnoses were made based on the Structured Clinical Interview for DSM-IV. Study 2 examined self-directed passive-aggressive behaviour as a mediator of the relationship between distorted self-monitoring and dysfunctional self-evaluation and self-reported depressive symptoms in 200 undergraduate Psychology students.

**Results:**

Compared to outpatients without depressive disorders, outpatients with depressive disorder reported significantly more self-directed passive aggression (*d* = 0.51). Furthermore, Study 2 verified self-directed passive-aggressive behaviour as a partial mediator of the relationship between dysfunctional attitudes (*ab*_*cs*_ = .22, 95%-CI: .14, .31), attributional style (*ab*_*cs*_ = .20, 95%-CI: .13, .27), ruminative response style (*ab*_*cs*_ = .15, 95%-CI: .09, .21) and depressive symptoms.

**Conclusion:**

Self-directed passive-aggressive behaviour partially mediates the association between distorted self-monitoring and dysfunctional self-evaluation with depressive symptoms. Future longitudinal studies need to examine a potential causal relationship that would form a base to include interventions targeting self-directed passive-aggressive behaviour in prevention and treatment of depression.

**Trial registration:**

Both studies were preregistered at the German Clinical Trials Register (DRKS00014005 and DRKS00019020).

## Introduction

Depressive disorders are severe mental illnesses associated with high societal [1] and individual burden [2, 3]. Main symptoms of depressive episodes are sad mood, diminished interest in activities, and fatigue or low levels of energy, lasting for at least two weeks [4]. Cognitive behavioural theories explain the aetiology of depression by diathesis-stress models with dysfunctional attitudes [5–8], rumination [9–13] and attributional style [14–16] as major psychological factors for the onset and persistence of depressive disorders. Lifetime prevalence of depressive disorders is high, ranging from 11.6% [17] to 17.1% in the German general population [18]. Ten to 17% of individuals with depressive disorders develop a chronic course [19], spending approximately 20.8% of their lifetime in depressive episodes [20]. Although cognitive behavioural psychotherapy for depression is effective in the short-term, 54% of initial responders relapse in a 2-year period after treatment [21] making the identification of other factors associated with the onset, persistence and—even more important—relapse of depressive episods an important research goal.

Self-directed aggression may constitute such a factor. One symptom of major depressive episodes are suicide ideations, plans and attempts [4]. Other forms of self-directed aggressive behaviour (i.e., non-suicidal self-harming behaviour) are not a symptom of depression per se, however, individuals with depressive disorders are at higher risk for these behaviours compared with other mental disorders [22] and depressive symptoms constitute a well-evidenced risk factor for deliberate self-harming behaviour [23]. In this regard, self-directed aggressive behaviour describes any behaviour intended to harm oneself in active or passive ways [24]. Self-directed *active* aggressive behaviour is defined as an active engagement in self-harm (e.g., cutting oneself, self-punishment; [25]), whereas self-directed passive-aggressive behaviour is defined as harmful inactivity [e.g., omission of one´s own needs or reduced self-reward; [26]]. The link between self-directed aggression and depression may be explained by the self-control model of depression [27], which is based on Kanfer's [28] behavioural self-control model. According to the self-control model of depression, depressive symptoms are a result of a maladaptive feedback loop of dysfunctional *self-monitoring* and distorted *self-evaluation*. Self-monitoring encompasses the observation of one’s own behaviour (including antecedences and consequences). In depression, this process tends to be dysfunctional by attending only to negative events and focus on (negative) short-term consequences and thereby neglecting potential future positive effects. Self-evaluation consists of the comparison between the information obtained from self-monitoring and internal standards [29]. In depressed individuals, self-evaluation is assumed to be inaccurate by stringent and rigid self-evaluation standards. Negative self-evaluation leads to reduced self-reward (self-directed passive-aggressive behaviour) and increased self-punishment (self-directed active-aggressive behaviour), and in turn to the onset and persistence of depressive symptoms.

One can also interpret the psychological factors (i.e., rumination, dysfunctional attitudes, and negative attribution style) of the diathesis-stress model within this framework: Rumination as obsessional thinking involving excessive, repetitive thoughts or themes that interfere with other forms of mental activity represents a form of dysfunctional self-monitoring [30, 31]. Dysfunctional attitudes that induce negative thoughts about the self, others, and the future [32] and negative attributional style as attributing negative events as being internal, stable and global [33] contribute to a distorted self-evaluation within the feedback loop. In line with this notion, correlations between self-directed active aggression with depressive symptoms, rumination [34, 35], dysfunctional attitudes [36, 37], and negative attributional style [38–40] have been demonstrated multiple times.

Evidence is scarcer on self-directed passive aggression. A recent study by Schanz et al. [41] found a medium-sized association between self-directed passive-aggressive behaviour and depressive symptoms in an inpatient sample. More severe depressive symptoms were associated with higher levels of passive-aggressive behaviour. In a joint model, depressive symptoms and somatization but not anxiety symptoms explained a unique proportion of variance in self-directed passive-aggressive behaviour. This was in line with the assumption that self-directed passive-aggressive behaviour may show associations with general psychological symptom burden and other symptom categories than depression as these share transdiagnostic cognitive risk factors [42]. However, the unique amount of variance accounted for by depression was the largest supporting its particular importance for depression. Yet, results from this study did not allow for conclusions on self-directed passive-aggressive behaviours for specific diagnoses categories as standardized clinical interviews could not be performed within the clinic setting. Therefore, analyses remained limited to symptom severity irrespective of diagnosis categories. In addition, the sample surveyed in this study was rather specific, as psychosomatic rehabilitations are often employed after long-term sick leaves and in the presence of comorbid somatic illnesses.

Given its potential role as risk factor and/or factor being associated with the persistence of depression by a lack of self-rewarding behaviour, intensified research efforts on the role of self-directed passive-aggressive behaviour in depression in different populations seem a promising avenue of identifying new prevention and treatment options for depressive disorders that are much needed considering the enormous burden of depression [19–21, 43].

### Study aims

Study 1 aims to determine whether the correlation between self-directed passive-aggressive behaviour and depressive symptoms found in inpatients [41] holds-up in patients seeking outpatient psychotherapy. Even more important, Study 1 investigates the hypothesis that depressed patients diagnosed with a standardized clinical interview report higher levels of self-directed passive-aggressive behaviour than patients with other mental disorders.

Based on the self-control model of depression, Study 2 aims to test the assumptions that self-directed passive-aggressive behaviour is associated with dysfunctional self-monitoring (rumination) and self-evaluation (dysfunctional attitudes and negative attributional style) processes and that it mediates their association with depressive symptoms in a sample of undergraduate Psychology students. Students were chosen as they are exposed to significant stressors [44] and aged around the peak onset of depressive disorders [45]. Additionally, Study 2 examines whether self-directed passive-aggressive behaviour accounts for a unique amount of variance in depressive symptoms when controlling for the described cognitive factors.

## Methods of study 1

### Participants and procedure

To examine the association between self-directed passive-aggressive behaviour and depression in a sample of patients seeking outpatient treatment, Study 1 recruited patients from the Centre for Cognitive-Behaviour Therapy at the Saarland University and the Institute for Postgraduate Studies in Psychotherapy Saarbruecken (preregistration at German Clinical Trials Register: DRKS00014005). Adult patients (age ≥ 18 years) were asked for participation in the study after their first consultation. According to the declaration of Helsinki [46], all participants gave written informed consent. The study was approved by the Ethic Committee of Saarland University. Diagnoses were based on the structured clinical interview for mental disorders for DSM-IV axis I [SCID-I; [47]]. Additionally, the participants completed the measures. After 12 sessions of outpatient treatment, a follow-up assessment will take place to analyse the interaction between symptom change and self-directed passive-aggressive behaviour during psychotherapy. To date, the follow-up is not completed and negatively impacted by the COVID-19 pandemic that delayed many treatments. If follow-up completions will be sufficient, results will be published elsewhere. Initially, 251 patients agreed to participate. For 31 patients the SCID-I interviews did not reveal any axis I disorder. Therefore, these patients were excluded from all subsequent analyses. For sample characteristics of the remaining 220 patients, see Table 1.

**Table 1:** Descriptive sample characteristics for Study 1

	Depression group	Depression only group (subgroup)	Control group
*n*	140	50	80
% female	67.9	64.0	65.0
Age *M* (*SD*, years)	40.01 (12.70)	41.50 (12.92)	36.16 (13.15)
Age range	18–65	22–65	18–76
BDI-II *M* (*SD*)	26.97 (10.35)	27.12 (9.96)	18.36 (8.90)
BSI *M* (*SD*)	1.42 (0.64)	1.33 (0.58)	1.02 (0.58)
TPA-SD *M* (*SD*)	2.93 (0.84)	2.95 (0.82)	2.53 (0.72)
One comorbidity (%)	37.9	-	21.3
Two comorbidities (%)	21.4	-	5.0
Three comorbidities (%)	2.9	-	1.3
Four comorbidities (%)	1.4	-	0
Five comorbidities (%)	0.7	-	0
Anxiety disorders (%)	42.9	-	53.8
Obsessive compulsive disorder (%)	7.1	-	13.8
PTSD (%)	6.4	-	5.0
Somatoform disorder (%)	12.9	-	6.3
Adjustment disorder (%)	0.7	-	25.0
Others (%)	10.7	-	10.0

*M* Mean, *SD* Standard deviation. Beck Depression Inventory II; *BSI* Brief Symptom Inventory, *TPA-SD* Test of Passive Aggression – self-directed, *PTSD* Posttraumatic stress disorder

The ‘depression only group’ comprises those patients of the ‘depression group’ that are solely diagnosed with a depressive disorder

### Measures

SCID is a structured clinical interview based on diagnostic criteria of DSM [47, 48]. SCID has shown to be the gold standard for diagnosis of mental disorders (e.g., affective disorders, anxiety disorders, eating disorders; [49–51]). Given that the German version of the SCID-5-CV [52] was not available at the start of the present study, diagnoses were obtained using the German version of the SCID-I for DSM-IV [53]. SCID-I interviews were conducted by the first author (clinical psychologist, master level) as well as by trained and supervised students of clinical psychology (bachelor level). The SCID-I was found to show good validity and interrater agreement reflected in *Cohen’s kappa* ≥ 0.70 [54, 55].

The Beck-Depression Inventory-II (BDI-II; [56]) is a self-reporting questionnaire comprising 21 items to assess depressive symptoms based on DSM-IV criteria [48]. Higher item scores indicate stronger depressive symptoms. The BDI-II is a reliable and valid instrument measuring depressive symptom severity [57, 58]. The BDI-II was found to be a reliable and valid assessment of depressive symptoms [59]. In Study 1, the BDI-II exhibited an excellent internal consistency (*α* = 0.90, *n* = 213).

The Test of Passive Aggression (TPA; [41]) is a self-reporting instrument to assess self-directed (TPA-SD) and other-directed passive aggression (TPA-OD). The validity of the TPA-SD has been demonstrated in a previous study with measures for self-directed aggression [60], impulsivity [61], and personality traits [e.g., neuroticism [62, 63]]. TPA-SD demonstrated good internal consistency in the current sample (*α* = 0.86; *n* = 220).

The Brief Symptom Inventory (BSI; [64]) is a short version of the Symptom Checklist 90 Revised [65, 66] assessing the severity of psychopathological symptoms. The 53-item self-reporting questionnaire assesses nine symptom scales (e.g., somatization, anxiety, and obsessive-compulsion), which can be summed up to a global severity index (GSI) reflecting general symptom burden [67]. The BSI was found to be reliable and valid in previous studies [68, 69]. In the presented study the GSI demonstrated excellent internal consistency (*α* = 0.95, *n* = 215).

### Statistical analyses

All analyses were performed using IBM SPSS Statistics version 25 [70]. Bivariate relationships between self-directed passive aggression (TPA-SD), depressive symptoms (BDI-II), and global symptom severity (BSI) were analysed using Pearson correlation coefficients (*r*). To control for the association between self-directed passive aggression with depression and general psychopathology, a multiple regression analyses including GSI score as predictor variable was conducted. An ANOVA with depression group versus control group as between-subject factor was performed to examine the hypothesis that patients with depressive disorders report more self-directed passive aggression than controls. Inclusion criteria for the depression group were unipolar affective disorders (including recurrent depression and dysthymia). To control for effects of comorbidity on self-directed passive aggression, the same analysis was rerun excluding all patients of the depression group with comorbid disorders (hereinafter referred to as ‘depression only’ group). Both analyses were repeated with age and gender being controlled for.

## Results of study 1

### Association between self-directed passive-aggressive behaviour and depressive symptoms

In line with our expectations, self-directed passive-aggressive behaviour and depressive symptoms were significantly correlated, *r* = 0.56, *p* < 0.001. This correlation remained stable after controlling for global symptom severity (see Table 2).

**Table 2:** Multiple regression model for self-directed passive-aggressive behaviour (TPA-SD) in Study 1

	*β*	*t*	*p*	*r*
BSI	0.17	1.76	0.079	0.51
BDI-II	0.42	4.30	< 0.001	0.56

*BDI-II* Beck Depression Inventory II, *BSI* Brief Symptom Inventory, *TPA-SD* Test of Passive Aggression—self-directed

For all zero-order correlations *p* < 0.001. F_Modell_ (2, 209) = 50.30; *R*^*2*^ = 0.33; *p* < 0.001

### Group differences in self-directed passive-aggressive behaviour

An ANOVA with group (depression group vs. control group) as between-subject factor and self-directed passive aggression as dependent variable revealed a significant difference between the two groups [*F*(1, 218) = 13.23; *p* < 0.001; *d* = 0.51] with depressed patients reporting more self-directed passive aggression than controls. Moreover, a significant difference in self-directed passive aggression was also evident between the depression only group and the control group [*F*(1, 128) = 9.74; *p* = 0.002; *d* = 0.57]. Both effects remained significant after controlling for age and gender.

## Methods of study 2

### Participants and procedure

Participants of Study 2 (preregistered at German Clinical Trials Register: DRKS00019020) were adult (age ≥ 18 years) undergraduate (mostly freshmen) Psychology students at Saarland University. The participants received course credits for their participation. Data were collected using the online platform *SoSci Survey* [71] as we considered a paper-and-pencil assessments during course time as inappropriate due to data protection concerns. After giving written informed consent according to the Declaration of Helsinki [46], the participants completed the measures. In total, 200 students completed the online survey (for descriptive statistics see Table 3).

**Table 3:** Descriptive sample characteristics in Study 2

	Sample characteristics
*n*	200
% female	77.0
Age *M* (*SD,* years)	21.76 (4.55)
Age range (years)	18 – 46
BDI-II *M* (*SD*)	8.60 (7.73)
BSCL *M* (*SD*)	0.65 (0.51)
TPA-SD *M* (*SD*)	2.44 (0.64)
DAS-SF *M* (*SD*)	58.09 (15.88)
CSQ-SF *M* (*SD*)	202.44 (20.84)
RSS *M* (*SD*)	44.87 (11.30)

Beck Depression Inventory II; *BSCL* Brief Symptom Checklist, *TPA-SD* Test inventory of passive aggression—self-directed, *DAS-SF* Dysfunctional Attitude Scale – Short Form A, *CSQ-SF* Cognitive Style Questionnaire – Short Form, *RSS* Ruminative Response Scale

### Measures

The Cognitive Style Questionnaire (CSQ; [72]) is a self-reporting questionnaire assessing different attributional styles based on the hopelessness theory [14, 72, 73]. The German short form of the CSQ (CSQ-SF) used in this study comprises 72 items and consists of three scales (internality, globality, and stability of attribution), which can be summarized to a global score [74]. The scale was found to be sufficiently reliable and valid [73, 75]. In the current sample, internal consistency of the global score was good (*α* = 0.83).

The Rumination Response Scale (RSS; [9, 76]) is a subscale of the Response Style Questionnaire (RSQ) and assesses rumination according to the response-style theory [10, 58]. The RSS consists of 22 items with higher scores indicating a stronger ruminative response style. The RSS was found to demonstrate good reliability and validity in different studies [77, 78]. In Study 2, the RSS demonstrated excellent internal consistency (*α* = 0.91).

Based on Beck’s cognitive theory the Dysfunctional Attitude Scale [DAS; [79, 80]] assesses dysfunctional attitudes using 40 items. The German short form of the DAS (DAS-SF) exists in two parallel versions (Form A and B; [79, 81]). Each version consists of 18 items of the original DAS. The scale was shown to be reliable and valid in previous studies [80, 82]. In this study, short form A was used, which showed good internal consistency (*α* = 0.88).

The Brief Symptom Checklist (BSCL; [83]) is a revised version of the BSI used in Study 1 [65, 66]. Both questionnaires differ with respect to their item order only. The BSCL was found to be a reliable and valid instrument for the assessment of psychopathological symptom burden in different populations [84, 85]. In the current sample, the GSI showed excellent internal consistency (*α* = 0.96).

As in Study 1, depressive symptoms were assessed using the BDI-II and self-directed passive aggression using the TPA-SD, with good internal consistencies for the BDI-II (*α* = 0.90) and acceptable internal consistency for the TPA-SD (*α* = 0.78). Reliability and validity of those scales has also been shown in previous studies (see Measures in Study 1).

Additionally, trait-anger [86, 87], trait-anxiety [88], trait-shame [89] and sense of coherence [90] were assessed. Findings on these constructs will be reported elsewhere.

### Statistical analyses

All analyses were performed using IBM SPSS Statistics 25 [70]. Associations between self-directed passive aggression (TPA-SD), depressive symptoms (BDI-II), global symptom severity (BSCL) as well as cognitive factors of depression [ruminative response style (RSS), dysfunctional attitudes (DAS-SF), and dysfunctional attributional style (CSQ-SF)] were analysed using Pearson correlation coefficients (*r*) and multiple regression analyses. Moreover, mediation hypotheses were tested using the SPSS macro Process [91]. In accordance to Preacher and Kelley [92] indirect effects were completely standardized (*ab*_*cs*_).

## Results of study 2

### Association between self-directed passive-aggressive behaviour and depressive symptoms

Self-directed passive-aggressive behaviour was strongly associated with depressive symptoms, *r* = 0.54, *p* < 0.001. This association remained significant after controlling for global symptom severity (see Table 4).

**Table 4:** Multiple regression models for self-directed passive-aggressive behaviour (TPA-SD) in Study 2

	*β*	*t*	*p*	*r*
BSCL	0.22	2.18	0.030	0.51
BDI-II	0.37	3.73	< 0.001	0.54

*BDI-II* Beck Depression Inventory II, *BSCL* Brief Symptom Checklist, *TPA-SD* Test inventory of passive aggression—self-directed

For all zero-order correlations *p* < 0.001. *F*(2, 197) = 44.01; *R*^*2*^ = 0.31; *p* < 0.001

### Association between self-directed passive-aggressive behaviour and cognitive factors

Self-directed passive aggression was positively associated with all cognitive factors of depression (see Table 5). In a joint multiple regression model, all cognitive factors accounted for a unique amount of variance in self-directed passive aggression (see Table 5).

**Table 5:** Multiple regression model for self-directed passive aggression in Study 2

	*β*	*t*	*p*	*r*
RSS	0.24	3.53	0.001	0.45
CSQ-SF	0.18	3.47	0.024	0.48
DAS-SF	0.36	5.00	< 0.001	0.54

*DAS-SF* Dysfunctional Attitude Scale—Short Form A, *CSQ-SF* Cognitive Style Questionnaire—Short Form, *RSS* Ruminative Response Scale

For all zero-order correlations *p* < 0.001. *F*(3, 196) = 38.40; *R*^*2*^ = 0.37; *p* < 0.001

### Unique association of self-directed passive-aggressive behaviour with depressive symptoms

Depressive symptoms were significantly associated with all cognitive factors (see Table 6). When accounted for the influence of cognitive factors in a multiple regression model, self-directed passive aggression still explained an incremental proportion of variance in depressive symptoms [*F*(1, 195) = 15.12; *ΔR*^*2*^ = 0.04; *p* < 0.001; see Table 6].

**Table 6:** Multiple regression model for the prediction of depressive symptoms in Study 2

		*β*	*t*	*p*	*r*
Model 1	RSS	0.48	7.68	< 0.001	0.62
	CSQ-SF	0.12	1.79	0.075	0.46
	DAS-SF	0.20	3.07	0.002	0.47
Model 2	RSS	0.42	6.86	< 0.001	0.62
	CSQ-SF	0.08	1.12	0.251	0.46
	DAS-SF	0.12	1.70	0.089	0.47
	TPA-SD	0.25	3.89	< 0.001	0.54

*BDI-II* Beck Depression Inventory II, *TPA-SD* Test of Passive Aggression—self-directed, *DAS-SF* Dysfunctional Attitude Scale—Short Form A, *CSQ-SF* Cognitive Style Questionnaire—Short Form, *RSS* Ruminative Response Scale

For all zero-order correlations *p* < 0.001. *F*_*Model* 1_(3, 196) = 53.36; *R*^*2*^ = 0.45; *p* < 0.001; *F*_*Model 2*_(4, 195) = 46.68; *R*^*2*^ = 0.49; *p* < 0.001

### Self-directed passive-aggressive behaviour as a mediator

As expected, self-directed passive aggression mediated the relationship of dysfunctional attitudes (*ab*_*cs*_ = 0.22, 95%-CI: 0.14, 0.31), attributional style (*ab*_*cs*_ = 0.20, 95%-CI: 0.13, 0.27), and ruminative response style (*ab*_*cs*_ = 0.15, 95% CI: 0.09, 0.21) with depressive symptoms. However, all cognitive factors showed significant direct effects (*p* < 0.001).

## Discussion

Study 1 supported the hypothesis that depressed patients diagnosed with a standardized clinical interview [53] report higher levels of self-directed passive-aggressive behaviour than patients with other mental disorders. In line with previous findings from our group [41], both studies confirmed a strong correlation between self-directed passive-aggressive behaviour and depressive symptoms as a continuous outcome. In multiple analyses, this association remained robust when gender, age, general psychopathology, and psychological factors for the onset and persistence of depression were controlled for. Furthermore, mediation analysis in Study 2 showed that self-directed passive-aggressive behaviour serves as a partial mediator for the relationship between these factors and depression.

### Bivariate association between depressive symptoms and self-directed passive aggression

According to the self-control model of depression, insufficient self-reward (a component of self-directed passive aggression) is a major cause of the development and persistence of depressive disorders [27]. The results of Study 1 support this hypothesis by showing that patients with depressive disorder had significantly higher scores on an inventory of self-directed passive aggression than patients with other mental disorders. Beyond previous findings [41], the current study was able to demonstrate that this finding also holds when depression is assessed using a clinical-administered interview. Thereby, our findings suggest that self-directed passive-aggressive behaviour may constitute a risk factor, symptom or correlate showing a particularly strong association with depression. This notion is also supported by the finding that the differences between patients with depression and other mental disorders remained stable even when controlling for general mental distress highlighting that self-directed passive-aggressive behaviour not simply reflects severe levels of mental distress.

Moreover, both studies provided further evidence for the hypothesis that there is a specific association between self-directed passive aggression and self-reported depressive symptoms. Together with our previous findings [41], these results underline the robustness of the association among diverse samples (i.e., inpatient and outpatient samples, student samples). These findings are also in line with research on self-directed active aggression that demonstrated robust association with self-reported depressive symptoms and clinician-made diagnoses [93].

### Self-directed passive-aggressive behaviour and cognitive factors of depression

Based on the self-control model of depression [27] and previous studies demonstrating associations between self-directed active-aggressive behaviour and ruminative response style [34, 35], attributional style [39, 40] as well as dysfunctional attitudes [36, 37], we hypothesized that these factors are correlated with self-directed passive aggression. Findings of Study 2 found these associations, thereby supporting the notion that dysfunctional self-monitoring (rumination) as well as distorted self-evaluation (dysfunctional attitudes and attributional style) contribute to self-directed passive aggression. Furthermore, the potential relevance of self-directed passive aggression for depression was supported by a unique amount of variance explained by self-directed passive aggression in depressive symptoms (even after controlling for cognitive factors of depression). Thereby, our findings suggest that self-directed passive aggression may capture a component of depressive symptoms that is not yet adequately addressed by standard cognitive factors of depression (e.g., [5]).

To further examine the relationship between self-directed passive-aggressive behaviour and self-reported depressive symptoms, we examined self-directed passive aggression as a mediator of the relationship between cognitive factors and depressive symptoms; and were able to demonstrate a partially mediating effect of self-directed passive aggression.

### What links self-directed passive aggression and depression?

Our findings show the robustness of the relationship between self-directed passive aggression and depressive symptoms and at the same time, raise the question whether self-directed passive may constitute a risk factor for depression, a factor associated with persistent depressive episodes and/or a symptom or consequence of depression. Moreover, we cannot exclude that a third variable (not assessed in our study) accounts for the association even though cognitive risk factors for depression did not account for the relationship as shown in Study 2. Following the self-control model of depression [27] self-directed passive-aggressive behaviour as self-induced denial of reward could reflect the behavioural readout of dysfunctional self-monitoring and self-evaluation. Which itself makes dysfunctional self-monitoring and self-evaluation more likely and could potentially reflect a vicious circle. However, in order to investigate such a model longitudinal research and also experimental studies would be needed. Such studies should also examine the role of cognitive risk factors for depression as potential percussors of self-directed passive-aggressive behaviour and should investigate the bidirectional relationship between self-directed passive aggression and depressive symptoms as it has recently been done for rumination [94]. Based on these findings a comprehensive model integrating the concept of self-directed passive aggression within the framework of a diathesis-stress model of depression should be developed.

### Potential use of self-directed passive aggression in psychotherapy

Given that longitudinal studies would provide evidence for a crucial role of self-directed passive aggression for depression, the concept might hold the potential to advance prevention and/or treatment of depression. In psychotherapy self-directed passive-aggressive behaviour could be assessed (using the TPA as screening instrument [41]) and targeted by behavioural change techniques of cognitive behavioural therapy [94]. This might be of particular relevance as self-directed passive-aggressive behaviours could be overlooked by standard assessments and treatment. Therefore, future studies need to examine if standard treatments result in significant changes in self-directed passive-aggressive behaviour. Moreover, given the fact that self-directed passive aggression may also predict relapses, interventions targeting self-directed passive aggression may be included in relapse prevention modules and could be used to monitor individual relapse risk. However, the use of self-directed passive aggression in treatment and prevention of depression requires more research employing more elaborated designs. The current findings do not allow for inferences on the use of the concept in psychotherapy.

### Limitations and future directions

Several limitations need to be taken into account when interpreting the findings of our studies. Study 2 supports the hypothesis of self-directed passive aggression being a (partial) mediator of the relationship between cognitive correlates of depression and depressive symptom severity. However, the sample consisted of undergraduate Psychology students, with low levels of psychopathological symptoms [mean BDI-II < 14 (cut-off for minimal depression), mean BSCL < 0.68 (clinical cut-off for a mixed-gender student sample)]. Thus, the restriction of variance caused by the overall low symptom levels could have reduced external validity and generalizability of our results [95]. For the present study, the student sample was chosen as previous studies found incidences of 7% per year for major depression [96] in students. These high incidences are in line with a meta-analysis showing that 75% of mental disorders onset before the age of 25 [peak of onset for depression: 20.5 years; [45]] and might reflect exposure to stressors related to the transition from school to university [e.g., change of residency, making new friends, adjusting to demands at the university; [44]]. However, Psychology students from one university represent a highly selective sample that may bias our findings. Thus, future studies need to replicate our results in more diverse samples from the general population and, even more important, in diverse patient samples. However, the present study provides important hypotheses for more concise future research in these samples.

Additionally, due to the cross-sectional study design, the present studies cannot clarify if self-directed passive-aggressive behaviour is a symptom of depressive disorders or a risk factor for its development. With respect to self-directive active aggression, previous studies found evidence for both directions (aggression leads to depression [97, 98] vs. depression leads to aggression [99–101]), with small empirical evidence in favour of the depression leads to aggression assumption. To examine whether self-directed passive aggression should be included in prevention and treatment strategies of depression, longitudinal studies in high-risk samples are needed.

## Conclusion

We demonstrated that self-directed passive aggression was higher in depressed patients than in patients with other mental disorders when diagnoses were made using a standardized clinical interview. In two studies self-directed passive aggression was found to be significantly associated with self-reported depressive symptoms. Additionally, Study 2 demonstrated self-directed passive aggression to be a partial mediator of the relationship between cognitive factors of depression and depressive symptoms. Future studies need to extend the results of Study 2 to more diverse samples from the general population and clinical samples and should examine the longitudinal (and potentially causal) bidirectional relationship between self-directed passive aggression and depressive symptoms.

## Data Availability

The datasets generated during the current study are not publicly available due to the ongoing follow up of Study 1. The datasets supporting the conclusion of this article are available from the corresponding author on reasonable request.

## Abbreviations

BDI-II: Beck-Depression Inventory-II
BSI: Brief Symptom Inventory
BSCL: Brief Symptom Checklist
CSQ: Cognitive Style Questionnaire
CSQ-SF: Cognitive Style Questionnaire – Short Form
DAS: Dysfunctional Attitude Scale
DAS-SF: Dysfunctional Attitude Scale – Short Form
DSM: Diagnostic and Statistical Manual of Mental Disorders
PTSD: Post-Traumatic Stress Disorder
RSS: Rumination Response Scale
SCID: Structured Clinical Interview for DSM
TPA: Test of Passive Aggression
TPA-SD: Test of Passive Aggression – self-directed
TPA-OD: Test of Passive Aggression – other-directed
